# Sentinel Lymph Node Positivity by Tumour Grade in Older Women With Breast Cancer: A Cross-Verified Retrospective Cohort Study

**DOI:** 10.7759/cureus.115926

**Published:** 2026-09-08

**Authors:** Raafat Mady, Omar Mady, Maryam Nazim, Gary Boland, Zuhair Saidan

**Affiliations:** 1 Breast Surgery, Kettering General Hospital NHS Foundation Trust, Kettering, GBR; 2 Surgery, University of Liverpool School of Medicine, Liverpool, GBR; 3 General Practice, St Andrews House Medical Centre, Stalybridge, GBR; 4 Breast Surgery, Chorley and South Ribble Hospital, Chorley, GBR

**Keywords:** breast cancer in old women, breast cancer research, low risk breast cancer, omission of axillary surgery, sentinel lymph node (sln)

## Abstract

Background: The Choosing Wisely campaign and several randomised trials have questioned the routine use of sentinel lymph node biopsy (SLNB) in older women with early-stage, hormone receptor-positive, human epidermal growth factor receptor 2 (HER2)-negative breast cancer, given the low rate of axillary nodal involvement and its limited impact on adjuvant management in this population.

Objective: To determine the rate of SLNB positivity in women aged ≥70 years with Grade 1, T1-T2, estrogen receptor (ER)-positive, HER2-negative invasive breast cancer, to compare this rate against Grade 2 and Grade 3 disease of similar stage and receptor profile, and to assess whether the data support selective omission of SLNB in this specific subgroup.

Methods: We conducted a retrospective cohort study at a single UK breast unit. Patients who underwent SLNB between January 2015 and December 2020 were identified from hospital coding records and cross-linked, via hospital identifier, to a separate histopathology database, with age at surgery independently verified using linked admission dates rather than assumed from the histopathology record alone. Patients meeting Grade 1, T1-T2, ER-positive, HER2-negative criteria - including mucinous carcinoma and invasive ductal carcinoma (IDC) with a papillary component, given their comparably favourable low-grade biology - formed the primary study subgroup. Proportions are reported with 95% Wilson score confidence intervals.

Results: Of 420 patients identified via hospital coding, 323 tumours in 319 patients formed the final, age-verified analytic cohort after cross-linkage and per-tumour verification. Within the ER-positive/HER2-negative population (n=243), the primary study subgroup - Grade 1, T1-T2 disease - comprised 39 patients (32 T1, 7 T2). SLNB was positive in 3 of 39 patients (7.7%; 95% CI 2.7-20.3%). Restricted to the same T1-T2, ER+/HER2- criteria, SLNB positivity rose with tumour grade: 7.7% in Grade 1 versus 19.8% (95% CI 13.9-27.5%) in Grade 2 and 13.7% (95% CI 6.8-25.7%) in Grade 3. All 39 patients were followed up for a minimum of five years and a maximum of eight years, with no axillary or locoregional recurrences observed.

Conclusion: In women aged ≥70 years with Grade 1, T1-T2, ER-positive, HER2-negative breast cancer, SLNB positivity is low (7.7%) and notably lower than in Grade 2 or 3 disease of similar stage and receptor profile. These findings suggest that tumour grade should be incorporated into SLNB omission criteria, rather than relying on age and receptor status alone, and identify Grade 1 disease specifically as the most favourable candidate for selective SLNB omission. As a retrospective, single-arm estimate of nodal positivity rather than a comparative safety study, this work is best regarded as hypothesis-generating, and the case for omission should be confirmed in larger, prospective, multicentre cohorts with an appropriate comparator.

## Introduction

Breast cancer is the second most common cancer in the United Kingdom, and its incidence rises with age; older women make up a growing proportion of newly diagnosed cases [1]. Surgical management of the axilla has evolved substantially over the past four decades: early trials such as NSABP-04 and the Guy’s Hospital trial showed that extensive axillary surgery confers no survival advantage in clinically node-negative disease [2,3], paving the way for sentinel lymph node biopsy (SLNB), which offers comparable staging accuracy to axillary lymph node dissection (ALND) with substantially lower morbidity [4]. SLNB’s oncological validity was subsequently confirmed by the NSABP B-32 trial, establishing it as the standard of care for axillary staging [5], and axillary management has continued to de-escalate even among node-positive patients: the ACOSOG Z0011 and AMAROS trials showed that completion axillary dissection could be safely replaced by observation or axillary radiotherapy in selected sentinel-node positive patients, with less morbidity and no compromise in survival or regional control [6,7]. Selective SLNB omission in low-risk older patients represents a further, more conservative step in this same trajectory. The aim of this study was therefore twofold: first, to determine the rate of SLNB positivity in women aged ≥70 years with Grade 1, T1-T2, estrogen receptor (ER)-positive, human epidermal growth factor receptor 2 (HER2)-negative invasive breast cancer at our unit, benchmarked against Grade 2 and 3 disease of the same stage and receptor profile; and second, to demonstrate a reproducible, independently age-verified approach to constructing such an audit cohort. This question is not new. Older patients with small, node-negative, hormone receptor-positive tumours represent a particularly favourable population, in whom axillary nodal involvement is uncommon and adjuvant systemic therapy decisions are less frequently altered by nodal status. The Milan III and CALGB 9343 trials showed that omitting axillary surgery, or axillary staging altogether, did not compromise survival or regional control in older women with small, node-negative tumours [8,9], and the PRIME II trial showed a similarly favourable therapeutic ratio for omitting radiotherapy in older, ER-positive patients on endocrine therapy [10]. These findings underpinned the 2016 Choosing Wisely recommendation, endorsed by the Society of Surgical Oncology and the American Society of Breast Surgeons, that SLNB should not be performed routinely in women aged ≥70 years with early-stage, ER-positive, HER2-negative breast cancer [11].

More recent randomised evidence has extended this logic beyond the elderly population specifically: the SOUND trial found axillary surgery omission non-inferior to SLNB across a broader age range [12], and the INSEMA trial - the largest randomised study in this area, reporting primary results in 2025 - confirmed that omitting axillary surgical staging did not compromise invasive disease-free survival in clinically node-negative, early breast cancer treated with breast-conserving therapy [13]. A 2024 systematic review and meta-analysis focused specifically on older women with ER-positive/HER2-negative disease reached a concordant conclusion: SLNB results infrequently altered adjuvant treatment decisions in this population [14]. Despite this accumulating trial evidence, adoption of SLNB omission in routine UK practice remains variable, and institution-level data on the true rate of nodal positivity in eligible patients are limited. In auditing our own practice, we also identified a broader methodological issue relevant to single-centre retrospective audits of this kind: histopathology databases are rarely linked to an independently verifiable record of the patient’s age at the time of surgery, creating a risk that cohort totals are estimated rather than confirmed. We addressed this by cross-linking our histopathology data with hospital coding records containing admission dates, allowing age at surgery to be verified for every patient in the final analysis; the full approach is described in the Methods, under Data Sources and Cohort Verification.

This work was previously presented as a meeting abstract at the American College of Surgeons 2023, Dubai Chapter, September 2023.

## Materials and methods

Study design and setting

This was a single-centre retrospective cohort study conducted at the Central Lancashire Breast Unit, Lancashire Teaching Hospitals NHS Foundation Trust. Ethical approval was obtained in accordance with institutional policy for retrospective audit and service evaluation studies. This report follows the STROBE (Strengthening the Reporting of Observational Studies in Epidemiology) guidance for cohort studies [15].

Definitions

Tumour grade was assigned using the Nottingham (Elston-Ellis) modification of the Scarff-Bloom Richardson grading system, and “Grade 1” throughout this report refers specifically to this scale. ER positivity was defined per institutional and American Society of Clinical Oncology-College of American Pathologists (ASCO-CAP) convention as ≥1% tumour nuclei staining on immunohistochemistry (IHC); HER2-negative status was defined as IHC 0/1+, or IHC 2+ with non-amplified in situ hybridisation. SLNB positivity was defined per American Joint Committee on Cancer (AJCC)/Union for International Cancer Control (UICC) criteria; for the purposes of this audit, a sentinel node was classified as positive when macrometastasis (deposit >2 mm) was present. Isolated tumour cells and micrometastases were not separately categorised in this dataset and are not distinguished from macrometastatic disease in the results that follow; this is discussed further as a limitation.

Data sources and cohort verification

Two independent data sources were used and cross-linked by unique hospital identifier. The first was the breast unit’s prospectively maintained histopathology database, which records tumour grade, receptor status (ER, PR, HER2), histological subtype, SLNB result, completion of axillary clearance, adjuvant treatment, recurrence, and vital status, but does not itself record the date of surgery. The second was the Trust’s hospital coding/administrative database, which records Office of Population Censuses and Surveys Classification of Interventions and Procedures, Fourth Revision (OPCS-4) procedure codes, admission dates, and date of birth for every coded episode of care. Because age at the time of surgery is central to the eligibility criteria for this study, we used the coding database to independently verify age at surgery for each patient in the histopathology database, rather than relying on an assumed or undocumented age. For the small number of patients with bilateral disease (two histopathology entries under one hospital identifier), procedures were matched to admission episodes in chronological order; where both procedures shared a single admission date - the case for the majority of these patients - this matching was unambiguous. Records that could not be confidently matched between the two databases, or for which the computed age at surgery was below 70 years, were excluded from the final analytic cohort.

Patient selection

All patients aged ≥70 years at the time of surgery who were diagnosed with early invasive breast cancer and underwent SLNB at this institution between January 2015 and December 2020, and for whom age ≥70 years could be independently confirmed as described above, were included in the initial cohort. A specific subgroup was then selected based on the following histopathological inclusion criteria: T1 or T2 tumour stage; Grade 1 histology; ER-positive receptor status; and HER2-negative status. Patients with pure invasive ductal carcinoma (IDC, no special type) formed the majority of this subgroup. Grade 1 mucinous carcinoma and IDC with papillary features were also included, given their similarly favourable biology and low nodal involvement rates, provided they otherwise met the T1-T2, ER-positive, HER2-negative inclusion criteria.

Exclusion criteria

Patients were excluded if they had a diagnosis of invasive lobular carcinoma (ILC), pure ductal carcinoma in situ (DCIS), recurrent tumour in the ipsilateral breast, T3 or T4 disease, triple-negative phenotype, or HER2-positive tumours. In patients with multiple synchronous ipsilateral tumours, the higher grade was used as the determining histological grade. Two tumours recorded under an “Intermediate” grading convention, rather than the standard Grade 1-3 (Nottingham) system, could not be reliably reclassified and were excluded from grade-stratified analysis.

Follow-up

All 39 patients in the study subgroup were followed up clinically and radiologically from the date of surgery, with a minimum follow-up period of five years and a maximum of eight years. Recurrence status (axillary, locoregional, or distant) and vital status were recorded from the histopathology database and cross-checked against clinical records at last follow-up. A formal, protocolised imaging surveillance schedule beyond routine clinical and radiological follow-up was not separately specified in the dataset.

Statistical analysis

Results were tabulated, and descriptive statistical analysis was performed. Positivity rates were expressed as proportions with percentages, alongside 95% confidence intervals (CIs) calculated using the Wilson score method to reflect the precision of estimates derived from a small sample. To allow a fair comparison of SLNB positivity across tumour grades, the Grade 2 and Grade 3 comparator groups were restricted to the same T1-T2, ER-positive, HER2-negative criteria applied to the Grade 1 study subgroup. Grade-stratified positivity rates are reported descriptively, with non-overlapping point estimates and CIs used to characterise the gradient; no formal inferential test (e.g. chi-square or trend test) was performed across grade strata, and this is noted as a limitation. Statistical analysis was performed using Python version 3.12 (Python Software Foundation, Wilmington, Delaware, USA) with the pandas and statsmodels libraries.

## Results

Cohort flow and data verification

Hospital coding records identified 420 unique patients (425 procedures, accounting for bilateral disease) who underwent SLNB at the unit between January 2015 and December 2020. Of these, 387 patients (392 procedures) were confirmed to be aged ≥70 years at the time of surgery by cross-referencing date of birth against the linked admission date. Histopathological data were successfully matched for 331 of these age-confirmed patients (343 tumours); histopathology could not be linked for the remaining 56 age-eligible patients (14.5% of the age-confirmed coding cohort), most likely reflecting incomplete overlap between the two source databases, and these patients could not be included in the receptor- and grade-stratified analyses that follow. Of the 343 matched tumours, 335 could be matched to a specific admission date, and of these, 323 tumours in 319 patients had a fully confirmed age ≥70 years at that specific surgical episode; this group of 323 tumours forms the analytic cohort for Table 1.

**Table 1:** Overall cohort and grade distribution within the ER-positive/HER2-negative population. Counts refer to tumours unless otherwise stated; a small number of patients contributed two tumours (bilateral disease). Of 343 tumours with matched histopathology, 323 (in 319 patients) had a fully confirmed age ≥70 years at that specific surgical episode and formed the analytic cohort shown here; the remaining 20 tumours could not be matched to a specific admission date or did not meet the age threshold at that specific episode (see "Cohort flow and data verification" section in the Results). ER: estrogen receptor; HER2: human epidermal growth factor receptor 2; SLNB: sentinel lymph node biopsy

Characteristic	n	Percentage (%)
Patients identified via hospital coding (SLNB, 2015-2020)	420	-
Confirmed aged ≥70 years at surgery	387	92.1% of coding cohort
Linked histopathology available (analytic cohort, tumours)	323	83.5% of age-confirmed cohort
ER-positive/HER2-negative	243	75.2% of analytic cohort
Grade 1 (within ER+/HER2-, all T stages)	39	16.0%
Grade 2 (within ER+/HER2-, all T stages)	147	60.5%
Grade 3 (within ER+/HER2-, all T stages)	55	22.6%
Grade recorded as “Intermediate” (excluded from grade-stratified analysis)	2	0.8%

Study subgroup (Grade 1, T1-T2, ER+/HER2-, age ≥70)

Thirty-nine patients met all inclusion criteria for the primary study subgroup: 34 had pure IDC, three had mucinous carcinoma, and two had IDC with a papillary component. T1 disease was present in 32 patients (82.1%) and T2 disease in seven patients (17.9%). Baseline characteristics of this subgroup are summarised in Table 2.

**Table 2:** Baseline characteristics of the Grade 1 study subgroup (n=39). IDC: invasive ductal carcinoma

Characteristic	n (%) or Mean/Median (Range)
Patients, n	39
Age at surgery, years - mean (SD)	79.0 (5.8)
Age at surgery, years - median (range)	77.7 (70.0-89.6)
Aged 70-79 years	22 (56.4)
Aged ≥80 years	17 (43.6)
Invasive tumour size, mm - mean (SD)	15.9 (10.6)
Invasive tumour size, mm - median (range)	14 (3-50)
T1	32 (82.1)
T2	7 (17.9)
Histological subtype - IDC	34 (87.2)
Histological subtype - Mucinous carcinoma	3 (7.7)
Histological subtype - IDC with papillary component	2 (5.1)
Left	24 (61.5)
Right	15 (38.5)

SLNB outcomes

Overall, three of 39 patients (7.7%; 95% CI 2.7-20.3%) had a positive sentinel lymph node. Two of the three SLNB-positive patients had T1 disease (2/32, 6.2%; 95% CI 1.7-20.1%); one had T2 disease (1/7, 14.3%; 95% CI 2.6-51.3%). Thirty-six patients (92.3%) had a negative SLNB. The distribution of SLNB outcomes by T stage is shown in Table 3.

**Table 3:** SLNB outcomes by T stage in the Grade 1, ER+/HER2- study subgroup aged ≥70 years. ER: estrogen receptor; HER2: human epidermal growth factor receptor 2; SLNB: sentinel lymph node biopsy; CI: confidence interval

T Stage	SLNB Positive	SLNB Negative	95% CI (Positive Rate)
T1 (n=32)	2 (6.2%)	30 (93.8%)	1.7-20.1%
T2 (n=7)	1 (14.3%)	6 (85.7%)	2.6-51.3%
Total (n=39)	3 (7.7%)	36 (92.3%)	2.7-20.3%

SLNB positivity by tumour grade

To place the Grade 1 result in context, SLNB positivity was also calculated for Grade 2 and Grade 3 tumours meeting the same T1-T2, ER-positive, HER2-negative criteria (Table 4). SLNB positivity rose progressively and substantially, with increasing tumour grade: 7.7% in Grade 1 compared with 19.8% in Grade 2 and 13.7% in Grade 3. This gradient supports the biological plausibility of selecting Grade 1 disease specifically, rather than the broader ER+/HER2- population irrespective of grade, as the appropriate target for SLNB omission.

**Table 4:** SLNB positivity by tumour grade, restricted to T1-T2, ER-positive, HER2-negative disease. The n values in this table (39, 131, 51) are restricted to T1-T2 tumours only and are therefore lower than the corresponding all-T-stage grade counts in Table 1 (39, 147, 55): Table 1 reports each grade across all T stages within the ER-positive/HER2-negative population, whereas this table restricts Grade 2 and Grade 3 to the same T1-T2 subset used to define the Grade 1 study subgroup, to allow a fair, stage-matched comparison. Grade 1 n is identical in both tables because all Grade 1 tumours in this ER-positive/HER2-negative cohort were T1-T2. ER: estrogen receptor; HER2: human epidermal growth factor receptor 2; SLNB: sentinel lymph node biopsy; CI: confidence interval

Grade	n	SLNB Positive	95% CI
Grade 1	39	3 (7.7%)	2.7-20.3%
Grade 2	131	26 (19.8%)	13.9-27.5%
Grade 3	51	7 (13.7%)	6.8-25.7%

SLNB-positive patient characteristics

The three patients with a positive SLNB within the Grade 1 study subgroup are described in Table 5. Patient 1 had a T2 tumour in the left upper outer quadrant with three of six sentinel nodes positive; she underwent wide local excision with adjuvant breast, axillary, and supraclavicular fossa radiotherapy, without completion axillary clearance. Patient 2 had a T1 tumour with one of one sentinel nodes positive (macrometastasis); she underwent completion axillary clearance, which revealed no further nodal involvement (0/7 additional nodes), followed by mastectomy without radiotherapy. Patient 3 had a T1 tumour in the left upper outer quadrant with one of four sentinel nodes positive (macrometastasis); she underwent mastectomy without completion axillary clearance or radiotherapy.

**Table 5:** Characteristics of the three SLNB-positive patients within the Grade 1 study subgroup. T: tumour; SLN: sentinel lymph node; ALND: axillary lymph node dissection; WLE: wide local excision; RT: radiotherapy; SCF: supraclavicular fossa

Patient	T Stage	Location	SLN Result	ALND	Surgery/Adjuvant RT	Recurrence
Pt1	T2	L upper outer quadrant	3/6 positive (macro)	No	WLE; RT (breast/axilla/SCF)	No
Pt2	T1	L upper half, 12 o’clock	1/1 positive (macro)	Yes (0/7)	Mastectomy; no RT	No
Pt3	T1	L upper outer quadrant	1/4 positive (macro)	No	Mastectomy; no RT	No

Recurrence and survival

Recurrence and survival outcomes at last follow-up (minimum five years, maximum eight years from surgery) are summarised in Table 6. No axillary, locoregional, or distant recurrence was identified in any of the 39 patients in the study subgroup, including the three SLNB-positive patients. Nine patients (23.1%) had died by the last follow-up; none from breast cancer or its treatment.

**Table 6:** Recurrence and survival outcomes at last follow-up (n=39).

Outcome	n	% (of n=39)
Minimum follow-up duration, years	5	-
Maximum follow-up duration, years	8	-
Axillary or locoregional recurrence	0	0%
Distant recurrence	0	0%
Alive at last follow-up	30	76.9%
Deceased - unrelated cause	8	20.5%
Deceased - pancreatic cancer	1	2.6%
Deceased - breast cancer or treatment-related	0	0%

## Discussion

Summary of key findings

This retrospective cohort study of 39 patients aged ≥70 years with Grade 1, T1-T2, ER-positive, HER2-negative invasive breast cancer found an SLNB positivity rate of 7.7% (95% CI 2.7-20.3%), with no axillary or locoregional recurrences observed over a minimum follow-up of five years (maximum eight years). SLNB positivity in this Grade 1 subgroup was substantially lower than in Grade 2 (19.8%) or Grade 3 (13.7%) tumours of identical stage and receptor profile, indicating that grade carries meaningful additional information about nodal risk beyond T stage and receptor status alone in this older population. Taken together, these findings suggest that grade should be incorporated into SLNB omission criteria, rather than applying age and receptor status alone, and identify Grade 1 disease specifically - rather than the ER-positive/HER2-negative population as a whole - as the most defensible target for selective SLNB omission.

Comparison with existing literature

These findings sit within the substantial evidence base for axillary de-escalation summarised in the Introduction. In brief, CALGB 9343 and Milan III established that omitting axillary staging or surgery does not compromise outcomes in older women with small, node-negative tumours [8,9]; PRIME II showed a similarly favourable trade-off for omitting radiotherapy [10]; and the more recent SOUND and INSEMA trials extended this principle to omission of axillary surgery altogether across a broader age range [12,13]. The 2016 Choosing Wisely recommendation formalised the position that SLNB should not be performed routinely in women ≥70 years with early-stage, ER-positive, HER2-negative disease [11], and a 2024 meta-analysis in this same population found that SLNB results infrequently changed adjuvant treatment decisions [14]. Our grade-stratified positivity rates are broadly consistent with this literature and add institution-level, UK-specific data to a question that has so far been addressed largely through trial populations and pooled analyses.

Clinical implications

It is important to be precise about what this study can, and cannot, support. This is a single-arm, retrospective estimate of SLNB positivity in a defined subgroup; it demonstrates that nodal involvement is uncommon in Grade 1 disease, and less common than in Grade 2 or 3 disease of the same stage and receptor profile, but it does not, by itself, demonstrate the safety of an omission strategy, since no comparator arm in which SLNB was actually omitted - with outcomes tracked - was included. Applying the Choosing Wisely recommendation strictly to our Grade 1 subgroup would have avoided SLNB in all 39 patients; the three patients ultimately found to have nodal involvement received individualised adjuvant treatment, and none recurred during follow-up, which is reassuring but describes a small number of events and cannot substitute for a prospective comparison. We have therefore framed our conclusion as identifying Grade 1, T1-T2, ER-positive, HER2-negative disease as a rational candidate for selective SLNB omission, and as evidence that grade should inform omission criteria, rather than as a demonstration that omission is safe in this group. The manuscript does not propose a specific numerical positivity threshold below, which omission should be considered a formal policy; the trials and guidance summarised above generally regard positivity rates in the low single digits to low teens, in a population where nodal status has limited influence on adjuvant treatment, as consistent with an acceptable omission strategy, and our Grade 1 estimate (7.7%, with a wide CI extending to 20.3%) sits within that range but with limited precision. From a practical standpoint, omission of SLNB in this population would reduce operative time, anaesthetic duration, and postoperative morbidity - including seroma, nerve injury, and lymphoedema - considerations of particular relevance in older patients who may have significant comorbidities and for whom quality of life and functional independence are important outcomes in their own right. We also note, without a confirmed explanation, that Grade 3 positivity (13.7%) was lower than Grade 2 positivity (19.8%) in this cohort, despite Grade 3 tumours typically being considered more locally aggressive. Plausible explanations include the smaller Grade 3 sample size (n=51 vs. n=131) producing an imprecise estimate, differences in the balance of proliferative versus invasive behaviour across grades, or unmeasured heterogeneity within the Grade 2 group; we regard this as a descriptive observation warranting further study rather than a finding with an established biological explanation, and it does not alter our focus on Grade 1 disease as the subgroup with the lowest, most consistently favourable positivity rate.

Strengths and methodological considerations

A specific strength of this study is the independent, coding-verified approach to cohort construction, which we highlight as a methodological point of broader relevance to single-centre breast cancer audits. Our initial histopathology extract, taken in isolation, could not be independently verified against a documented age at surgery, and an earlier, unlinked summary of this cohort had reported a total of 434 patients and an SLNB positivity rate of 6.8% (37 T1 and seven T2 patients; 3/44 positive). When we cross-linked the histopathology data against hospital coding records containing verified admission dates, the confirmed age-eligible, histology-matched cohort was smaller (39 patients; 32 T1 and seven T2), and the recalculated positivity rate was 7.7% rather than 6.8%. The direction and overall magnitude of the finding - a low, single-digit-to-low-teens SLNB positivity rate in this subgroup - was unchanged by this correction, but the specific figures were not reproducible from the original, unlinked dataset. We present the verified figures throughout this report and would recommend that similar institutional audits incorporate independent age verification wherever possible.

Limitations

This was a single-centre, retrospective study with a small subgroup of 39 patients, which limits statistical precision, as reflected in the width of the 95% CIs reported throughout - the upper bound for the headline Grade 1 positivity estimate extends to 20.3%. As noted in the Methods, grade-stratified positivity rates were compared descriptively rather than with a formal statistical test (e.g., chi-square or trend test), and the apparent gradient should be interpreted with this in mind. Histopathological data could not be linked for 56 of the 387 age-eligible patients identified through hospital coding (14.5%); if these patients differ systematically from those with linked data, this could introduce selection bias that we cannot fully exclude with the data available. Isolated tumour cells and micrometastases were not separately categorised from macrometastatic disease in this dataset, and a formal, protocolised follow-up imaging schedule was not separately specified beyond routine clinical and radiological review; both limit the granularity with which nodal risk and surveillance intensity can be described. While all patients had a minimum of five years and a maximum of eight years of follow-up, the study was not powered to detect differences in survival or long-term locoregional control, and later recurrences beyond eight years were not captured. Two tumours recorded under a non-standard “Intermediate” grading convention were excluded from grade-stratified analysis, reflecting a degree of heterogeneity in historical grade recording at the unit. Finally, and most fundamentally, this single-arm design can estimate nodal positivity prevalence but cannot, on its own, establish the safety of SLNB omission; that question requires a comparator group in which axillary staging was genuinely omitted.

Future directions

Future work should include prospective, multicentre studies - ideally with a genuine comparator arm in which SLNB is omitted - to test the safety of omission directly in this subgroup, incorporating multivariate analysis of tumour size, lymphovascular invasion, multifocality, and comorbidity index, and using independent, coding-verified age and procedure data from the outset. The completed INSEMA and SOUND trials, together with ongoing evidence synthesis such as the 2024 meta-analysis by Daly and colleagues [14], will continue to refine eligibility criteria for axillary de-escalation in older women with favourable-biology breast cancer over the coming years.

## Conclusions

In this single-centre retrospective cohort study of women aged ≥70 years, the rate of SLNB positivity in Grade 1, T1-T2, ER-positive, HER2-negative invasive breast cancer was 7.7%, substantially lower than in Grade 2 (19.8%) or Grade 3 (13.7%) disease of identical stage and receptor profile, and no axillary or locoregional recurrences were observed over a minimum follow-up of five years (maximum eight years). These findings support incorporating tumour grade, alongside age and receptor status, into SLNB omission criteria, and identify Grade 1 disease specifically as the most favourable candidate subgroup for selective SLNB omission. As a retrospective, single-arm estimate of nodal positivity rather than a comparative safety study, this work is hypothesis-generating: larger, prospective, multicentre studies with independently verified cohort data, a genuine omission comparator, and multivariate analysis are required to confirm the safety of this de-escalation approach and to refine optimal patient selection criteria.
